# Intranasal Vaccine Using P10 Peptide Complexed within Chitosan Polymeric Nanoparticles as Experimental Therapy for Paracoccidioidomycosis in Murine Model

**DOI:** 10.3390/jof6030160

**Published:** 2020-09-02

**Authors:** Samuel Rodrigues Dos Santos Junior, Francenya Kelley Lopes da Silva, Lucas Santos Dias, Ana Camila Oliveira Souza, Marcelo Valdemir de Araujo, Leandro Buffoni Roque da Silva, Luiz R. Travassos, Andre Correa Amaral, Carlos P. Taborda

**Affiliations:** 1 Departamento de Microbiologia, Instituto de Ciências Biomedicas, Universidade de Sao Paulo, Sao Paulo, SP 05508-000, Brazil; dossantosdia@wisc.edu (L.S.D.); aolivei5@uthsc.edu (A.C.O.S.); marceloaraujo@usp.br (M.V.d.A.); leandrobr87@usp.br (L.B.R.d.S.); taborda@usp.br (C.P.T.); 2 Laboratorio de Nano & Biotecnologia, Departamento de Biotecnologia, Instituto de Patologia Tropical e Saúde Pública, Universidade Federal de Goias, Goiania, GO 74605-050, Brazil; francenya@hotmail.com (F.K.L.d.S.); amaral.nanobiotech@gmail.com (A.C.A.); 3 Departamento de Microbiologia, Imunologia e Parasitologia, Universidade Federal de Sao Paulo, Sao Paulo, SP 04023-062, Brazil; travassos@unifesp.br; 4 Departamento de Dermatologia, Instituto de Medicina Tropical de Sao Paulo, Faculdade de Medicina, Universidade de Sao Paulo, Sao Paulo, SP 05403-000, Brazil

**Keywords:** paracoccidioidomycosis, P10 peptide, nanovaccine, polymeric nanoparticles, antifungal therapy

## Abstract

Paracoccidioidomycosis (PCM) is a granulomatous fungal disease caused by the dimorphic fungal species of *Paracoccidioides*, which mainly affects the lungs. Modern strategies for the treatment and/or prevention of PCM are based on a Th1-type immune response, which is important for controlling the disease. One of the most studied candidates for a vaccine is the P10 peptide, derived from the 43 kDa glycoprotein of *Paracoccidioides brasiliensis*. In order to improve its immune modulatory effect, the P10 peptide was associated with a chitosan-conjugated nanoparticle. The nanoparticles presented 220 nm medium size, poly dispersion index (PDI) below 0.5, zeta potential of +20 mV and encapsulation efficiency around 90%. The nanoparticles’ non-toxicity was verified by hemolytic test and cell viability using murine macrophages. The nanoparticles were stable and presented physicochemical characteristics desirable for biological applications, reducing the fungal load and the usual standard concentration of the peptide from 4 to 20 times.

## 1. Introduction

Paracoccidioidomycosis (PCM) is a granulomatous disease caused by the thermo-dimorphic fungi belonging to the genus *Paracoccidioides*, endemic in Latin America, spreading from southern Mexico to the north of Argentina [1,2]. Although the affected organs are most frequently the lungs, PCM can also be a systemic disease [3]. 

This mycosis is characterized by skin and oral mucosa lesions, and the formation of granulomas, as a result of the lung infection and tissue damage. The liver and spleen can also be affected, leading to hepatosplenomegaly, which can cause organ malfunction [3,4]. Infection initiates when the host inhales fungal propagules dispersed in the atmosphere, which can then reach the alveoli. At the physiological temperature 37 °C, the fungus switches to a pathogenic yeast form [5,6]. 

The treatment of PCM is based on the patients’ clinical conditions and requires the use of chemotherapeutics, such as sulfamethoxazole trimethoprim, itraconazole and amphotericin B [3,7]. Some of these, however, can cause unwanted adverse side effects, for instance a long-term treatment or liver and kidney toxicity [8,9]. One of the alternative therapies that could be used to treat or prevent PCM is an immunomodulatory vaccine that increases the production of interferon gamma (IFN-γ) to stimulate a protective cell-mediated (Th1) immune response [10,11]. Different vaccine proposals, ranging from attenuated fungal particles to DNA-based vaccines have been investigated [12,13,14]. One of the most promising candidates for PCM is based on a 15-amino acid peptide (QTLIAIHTLAIRYAN) derived from the 43 kDa glycoprotein (GP43) of *Paracoccidioides brasiliensis* [15,16]. The P10 peptide stimulates the production of cytokines, such as IFN-γ, leading to a predominant Th1 immune response [15]. 

Although the P10 peptide is a promising vaccine candidate, a major drawback is its instability—similar to many other short peptides for in vivo application. An efficient approach used for the delivery of peptide vaccines actively being studied is the encapsulation or entrapment of the antigen within polymeric nanoparticles, which allows increased bioavailability of the immunogenic peptide [17,18]. 

Chitosan is a cationic, natural and low-cost polymer used to prepare these types of nanoparticles. The most striking feature of chitosan nanoparticles is their ability to be mucoadhesive. Given that PCM fungi are lined with mucus at the site of infection, this property of nanoparticles is essential for interactions in the respiratory tract, where degradation and displacement of particles takes place [19,20,21,22]. 

The objective of this study was to investigate an intranasal vaccine for the treatment of PCM using chitosan nanoparticles as a carrier for the P10 peptide.

## 2. Methods

### 2.1. P10-Chitosan Nanoparticles Preparation and Characterization

The nanoparticles were prepared according to modifications on the ionotronic gelation technique described by Calvo et al. [23,24]. Initially, 60 mg of chitosan (Chitosan Low Molecular Weight Sigma-Aldrich, St. Louis, MO, USA), deacetylation degree ≥ 75% and viscosity 20–300 cps (c = 1% in 1% of acetic acid) were diluted in 20 mL of ultrapure water and 174 μL of 0.1 M acetic acid (final concentration). The polymer was dissolved in a magnetic stirrer for approximately 1 h at room temperature. The pH of the solution was adjusted to 4.4 using NaOH 0.1 M and the final volume was adjusted to 30 mL with ultrapure water. For the sodium tripolyphosphate (TPP; Sigma-Aldrich, St. Louis, MO, USA) solution, 14 mg of TPP was dissolved in 14 mL of ultrapure water.

Complexed nanoparticles formation used 250 μL of P10 peptide at concentration of 20 μg/10 μL mixed with 1.13 mL of TPP solution. The TPP + P10 was slowly dripped into 2.12 mL of chitosan solution with magnetic stirring at 50–75 rpm for one hour at room temperature. The nanoparticles were then centrifuged for one hour at 13,200 rpm at 4 °C and the supernatant was collected and used for P10 association efficiency analysis. The empty nanoparticles were prepared following the same protocol but replacing the P10 peptide solution with 250 μL of ultra-pure water with 20% DMSO.

The pellet containing the empty or complexed nanoparticles was resuspended in 1 mL of PBS and stored at 4 °C for a week. 

Prior to administration, the nanoparticles were diluted in PBS to achieve concentrations of 1 μg/10 μL and 5 μg/10 μL of P10 peptide.

### 2.2. Physical-Chemical Characterization of Nanoparticles

The size (given as z-average), size distribution (given as polydispersity index (PDI) and surface charge (given as zeta potential) of the nanoparticles were determined using the Zetasizer Nano Zs equipment (Malvern Panalytical Ltd. Enigma Business Park. Grovewood Road. Malvern. WR14 1XZ. United Kingdom). All measurements were done at 25 °C. The association efficiency (AE) of P10 within chitosan nanoparticles was assessed by quantification of the non-complexed P10 peptide present in the supernatant using the Qubit™ Protein Assay Kit (Thermo Fisher Scientific, Waltham, MA, USA). The supernatant of the empty nanoparticles was used as the blank control in the measurements. AE was calculated using the Equation (1):

(1) $$\mathrm{AE}\;=\;\frac{\mathrm{Total}\;P10\;–\;\mathrm{Free}\;P10\;\mathrm{on}\;\mathrm{supernatant}}{\mathrm{Total}\;P10}\;\times \;100$$

All physiochemical characterizations were assesed using the complexed nanoparicles with the highest concentration of the P10 peptide 20 μg/10 μL.

### 2.3. Nanoparticles Safety Assays 

In order to evaluate the cytotoxicity of the empty or P10-complexed nanoparticles, cell viability was measured using the MTT 3-(4,5-dimethylthiazole bromide-2-yl)-2,5-diphenyltetrazolium bromide (Sigma-Aldrich, St. Louis, MO, USA) method and a hemolysis assay was performed according to [9].

#### 2.3.1. Hemolysis

Fresh blood was collected from healthy volunteers. Red blood cells (RBCs) were separated by centrifugation at 5000 rpm for 10 min, the supernatant was discarded, and the cells were washed three times with PBS. RBCs were then diluted in PBS (3:11). For the assay, 20 μL of RBC were distributed in the wells of a 96-well microplate containing 180 μL of empty or complexed P10 nanoparticles. Sterilized water was used as positive control (100% hemolysis) and 1× PBS was used for negative control (no hemolysis). P10-complexed nanoparticles were used at the concentrations of 0, 0.5, 1, 1.5, 2, 2.5 and 3 mg/mL. For empty nanoparticles, 2-fold serial dilutions of the initial concentration of nanoparticles (1×) were performed, where the dilutions (*n*) are represented as two to the power of *n* (2^−*n*^). Thus, the dilutions of the empty nanoparticles ranged from 1× (initial concentration after preparation) to 2^−6^×.

The samples were incubated for two or six hours at 37 °C and 5% CO_2_. After the incubation, the plates were centrifuged at 5000 rpm for 10 min at room temperature. The supernatant was transferred to another 96-well microplate and left at room temperature for 30 min to oxidize the hemoglobin of the lysed erythrocytes. The absorbance of the supernatants was then measured using a plate spectrophotometer reader at 540 nm.

The percentage of hemolysis was calculated using the following Equation (2),

(2) $$\%\;\mathrm{of}\;\mathrm{hemolysis}\;=\;\frac{\left(100\times \mathrm{AH}\right)}{\mathrm{AdH}2O}$$

where AH: Absorbance of sample, AdH_2_O: Mean of sample absorbances treated with distilled water.

#### 2.3.2. Cell Viability

Murine macrophage lineage J774.16 was also used to assess the cytoxicity of the nanoparticles. Macrophages were maintained in DMEM medium (Gibco, Thermo Fisher Scientific, Waltham, MA, USA) supplemented with 10% FBS (Fetal Bovine Serum, LGC Biotecnologia, Cotia, Sao Paulo, Brazil). First, 180 μL of DMEM supplemented with 10% FBS, containing nanoparticles with different amounts of P10 ranging from 400 μg to 0.19 μg were distributed in 96-well plates. Then, 20 µL of a suspension containing 5 × 10^4^ cells/mL was added to each well.

After 24, 48 and 72 h of incubation with the nanoparticles, 20 μL of 5 mg/mL MTT was added to each well and incubated at 37 °C and 5% CO_2_ for 4 h. The supernatants were then discarded and 100 μL of DMSO (dimethylsulfoxide, Sigma-Aldrich, St. Louis, MO, USA) was added to each well. Then, the absorbance of the supernatants was measured at 540 nm using a plate spectrophotometer reader. The percentage of live cells was calculated using the following Equation (3),

(3) $$\%\;\mathrm{of}\;\mathrm{live}\;\mathrm{cells}\;=\;\;\frac{\left(100\times \mathrm{Av}\right)}{\mathrm{ADMEM}}$$

where: Av: Absorbance of sample, ADMEM: Average absorbance of samples treated with DMEM (control).

### 2.4. In Vivo P10-Nanoparticles Vaccine Evaluation PBS

#### 2.4.1. Yeast

*P. brasiliensis* strain Pb 18 was maintained as yeast in Fava Neto solid medium. The yeasts were then transferred to the Brain Heart Infusion (BHI, BACTO^TM^, BD Franklin Lakes, NJ, USA) liquid medium supplemented with 4% fetal bovine serum (FBS), 4% glucose (DIFCO^TM^, BD Franklin Lakes, NJ, USA) and kept in a shaker at 37 °C and 150 rpm for five to seven days prior to infection. The yeast was then collected and washed three times using 1× and centrifuged at 3000 rpm for ten minutes. The yeast was resuspended in 5 mL of PBS and the viability of the fungal cells was assessed by counting in Neubauer’s chamber using the Janus green dye 1:1 (Sigma-Aldrich, St. Louis, MO, USA) and adjusted to 3 × 10^5^ yeast/50 μL. 

#### 2.4.2. Animals

Male BALB/c mice, free of pathogens, aged six to eight weeks, were used according to the Animal Use Ethics Committee: CEUA ICB #65/2016 (10/11/216). The animals were kept in the animal facility of the Microbiology Department of the Biomedical Science Institute and had access to water and food *ad libitum*. The animals were randomly organized accordingly: Group (i) positive control infected, without treatment (Pb 18); group (ii) infected and treated with a co-administered vaccine (empty nanoparticles + 20 μg/10 μL free-form of P10 peptide) (Nano + P10); group (iii) infected and treated with empty nanoparticles (0 μg); group (iv) infected and treated with the nanoparticles complexed with 1 μg/10 μL of P10 (1 μg); group (v) infected and treated with complexed nanoparticles with 5 μg/10 μL of P10 (5 μg); group (vi) infected and treated with nanoparticles complexed with 20 μg/10 μL of P10 (20 μg); group (vi) sham (uninfected and untreated).

#### 2.4.3. Intratracheal Infection

In order to simulate the infection caused by *P. brasiliensis*, BALB/c mice were infected with Pb18 yeasts through intratracheal inoculation. Briefly, animals were anesthetized intraperitoneally with 80 mg kg^−1^ of ketamine and 10 mg kg^−1^ of xylazine, and when the animals showed no reaction to any stimulus, indicating the anesthetic effect, an incision was made in the animal’s neck to expose the trachea in which 50 μL containing 3 × 10^5^ yeasts were inoculated. After infection, the mice were sutured and kept warm until full recovery.

#### 2.4.4. Immunization and Treatment

After 30 days of infection the mice received the nanoparticle vaccines intranasally, empty, complexed or co-administered, once a week for three weeks. Five microliters of the vaccine were administered into each nostril, resulting in 30 μL of vaccine per mouse at the end of treatment. Mice were euthanized 7 days after the last vaccination (51 days after infection was initiated).

#### 2.4.5. Evaluation of Treatment

The treatment efficacy was evaluated by counting colony forming units (CFU) from viable yeasts present in the lungs after 51 days of infection. 

After euthanasia, the lungs were aseptically removed, transversal sections of the tissue were randomly collected for histological processing, and the remaining tissue was weighted. The lungs were homogenized in 2 mL of PBS and 100 μL was plated in BHI agar supplemented with 4% (*v*/*v*) FBS, 4% (*v*/*v*) glucose, 5% (*v*/*v*) culture filtrate of *P. brasiliensis* strain 192 and 20 μg/mL gentamicin sulphate. After 21 days of incubation at 37 °C, colony forming units were counted.

#### 2.4.6. Cytokine Production Evaluation

For cytokine quantification, lung homogenate (500 μL) was aliquoted into microtubes, which contained 500 μL of protease inhibitor (Protease Inhibitor Panel (INHIB1) Sigma-Aldrich, St. Louis, MO, USA). The following recipe was used: Pepstatin A (P5318)—50 µg/mL, Benzamidine HCl (B6506)—50 mg/mL, N-Ethylmaleimide (E3876)—15.5 mg, EDTA (ED2SS)—1 mL−100 mM and distilled water q.s.p for 100 mL of protease inhibitor. 

Cytokine analysis was performed by Enzyme-Linked Immunosorbent Assay (ELISA) using commercial kits (BD OptEIA™, BD Franklin Lakes, NJ, USA) for the following cytokines IL-4, IL-6, IL-10, IL-12, and IFN-γ.

#### 2.4.7. Lung Histology

In order to evaluate the cellular preservation of the organ after treatment, lung transversal sections were fixed in 10% buffered formalin and embedded in paraffin using standard protocol. Histological slides were stained with haematoxylin and eosin (HE) for evaluation of the cellular structure of the organ.

### 2.5. Statistical Analysis

Analysis of variance (ANOVA) or student *t* test was performed followed by Tukey or Dunnett post-tests, using Graph Pad Prism 6. *p* values were considered significant when *p* ≤ 0.05 and error bars were used representing the standard error of the mean (SEM). 

## 3. Results

### 3.1. P10-Based Chitosan Nanoparticles Vaccine

The sizes and Zeta potential presented the expected values (Table 1) for this type of formulation, taking into account the preparation method and the materials used.

Both nanoparticles (empty and complexed) were smaller than 350 nm, presented a PDI below 0.5 indicating homogeneity in particle size, and a positive zeta potential ideal for muco-adhesiveness. The encapsulation efficiency of the peptide was above 90%. 

### 3.2. Cytotoxicity

**Figure 1:**
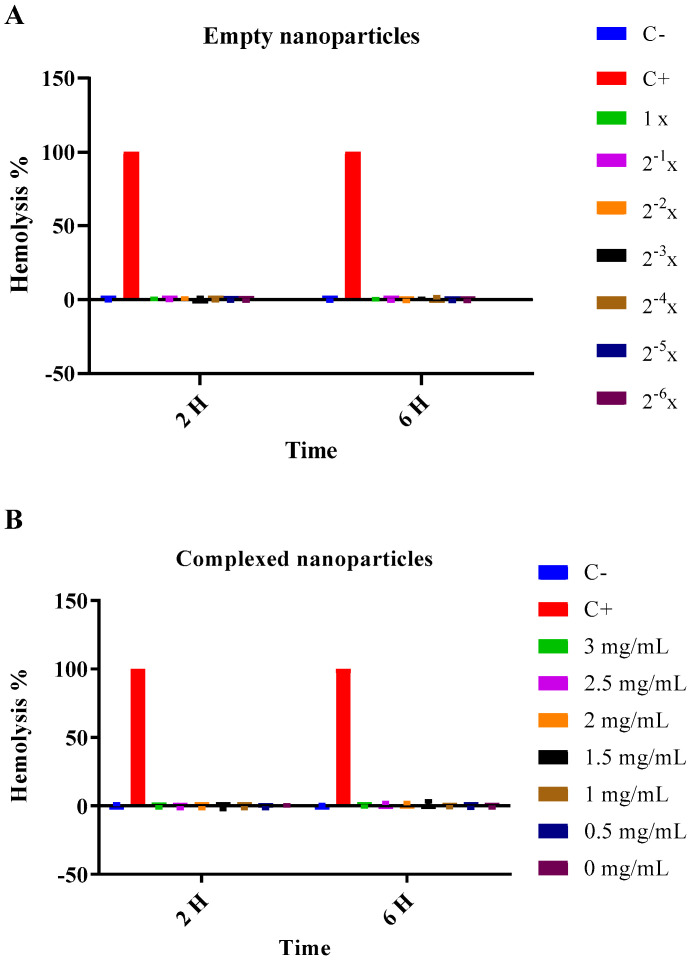
Hemolysis assay with empty or P10-complexed chitosan nanoparticles. Red Blood Cells were incubated with nanoparticles for 2 and 6 h and hemolysis was determined by absorbance measurement at 540 nm. Empty chitosan nanoparticles; (**A**) were serial diluted and concentrations ranged from 1× to 2^−6^×, with 1× being the initial concentration after preparation of the nanoparticles. The concentrations of P10-complexed chitosan nanoparticles; (**B**) varied from 3 mg/mL to 0 mg/mL. PBS was used as negative control (C−) (0% hemolysis) and autoclaved distilled water was used as positive control (C+) (100% hemolysis).

Neither the empty nor complex nanoparticles showed any hemolytic effect at 2 and 6 h (Figure 1) at the different concentrations analyzed. 

**Figure 2:**
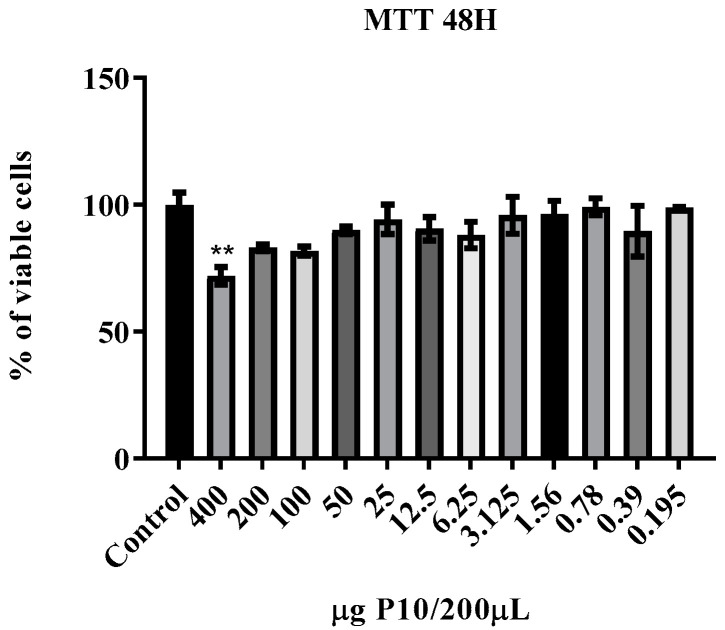

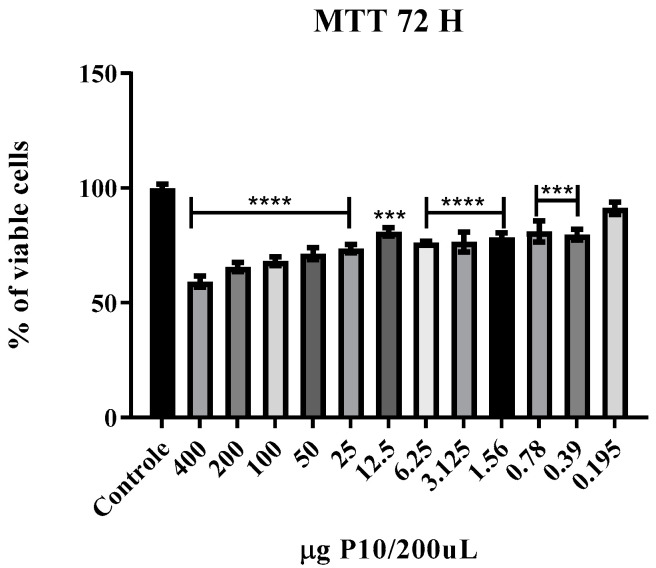
Cytotoxicity of P10-complexed chitosan nanoparticles in J774.16 macrophages with the aid of MTT colorimetric assay after 48 h and 72 h of co-incubation. At 48 h of incubation, only the concentration of 400 µg P10/200 µL showed toxicity to the cells (*p* ≤ 0.01) compared to control using only DMEM medium. At 72 h, a cytotoxicity pattern was shown for almost all the concentrations tested. Data are represented as mean SEM and were analyzed by ANOVA statistical test with ** *p* ≤ 0.01, *** *p* ≤ 0.001 and **** *p* ≤ 0.0001 in comparison to the control group (DMEM).

In the J774.16 cell viability assay, no cytotoxicity was observed in the first 24-h; in the 48-h period (Figure 2) only the highest concentration of P10 nanoparticles showed cytotoxicity. After 72-h of incubation, almost all concentrations of the P10 nanoparticles showed some degree of toxicity but none caused more than 50% cytotoxicity.

### 3.3. In Vivo Antifungal Efficacy of P10-Complexed Chitosan Nanoparticles

**Figure 3:**
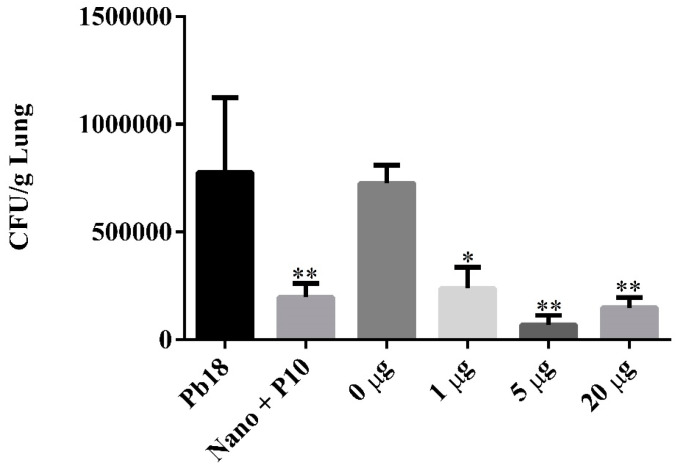
Fungal burden assessed by quantification of colony forming units per gram of lung tissue (CFU/g) in BALB/c mice after 51 days of *P. brasiliensis* infection. Mice were infected and randomly distributed in the following groups: Pb 18: non-treated; Nano + P10: co-administered vaccine (empty nanoparticles + 20 μg/10 μL free-form of P10 peptide); 0 μg: treated with empty nanoparticles; 1 μg: treated with P10-complexed nanoparticles containing 1 μg of P10/10 μL nanoparticles; 5 μg: treated with complexed nanoparticles containing 5 μg/10 μL of P10; 20 μg: treated with complexed nanoparticles containing 20 μg/10 μL of P10. Data are represented as mean SEM and were analyzed by ANOVA statistical test with * *p* ≤ 0.05 and ** *p* ≤ 0.01 in comparison to non-treated mice (Pb18). There was no statistical difference when comparing the groups Nano + P10, 1 μg, 5 μg and 20 μg.

The antifungal efficacy of the P10-complexed nanoparticles was evaluated 51 days after infection, corresponding to 30 days of infection and 21 days of therapy. In order to evaluate the therapeutic effectiveness, quantification of colony forming units (CFU) recovered from the lungs of the animals was performed. Both treatments with P10-complexed nanoparticles in different doses and empty nanoparticles mixed with the free P10 were effective in reducing the fungal load (Figure 3). P10-complexed nanoparticles were effective at even lower doses (1 and 5 μg).

### 3.4. Cytokine Production Induced by P10-Complexed Chitosan Nanoparticles

**Figure 4:**
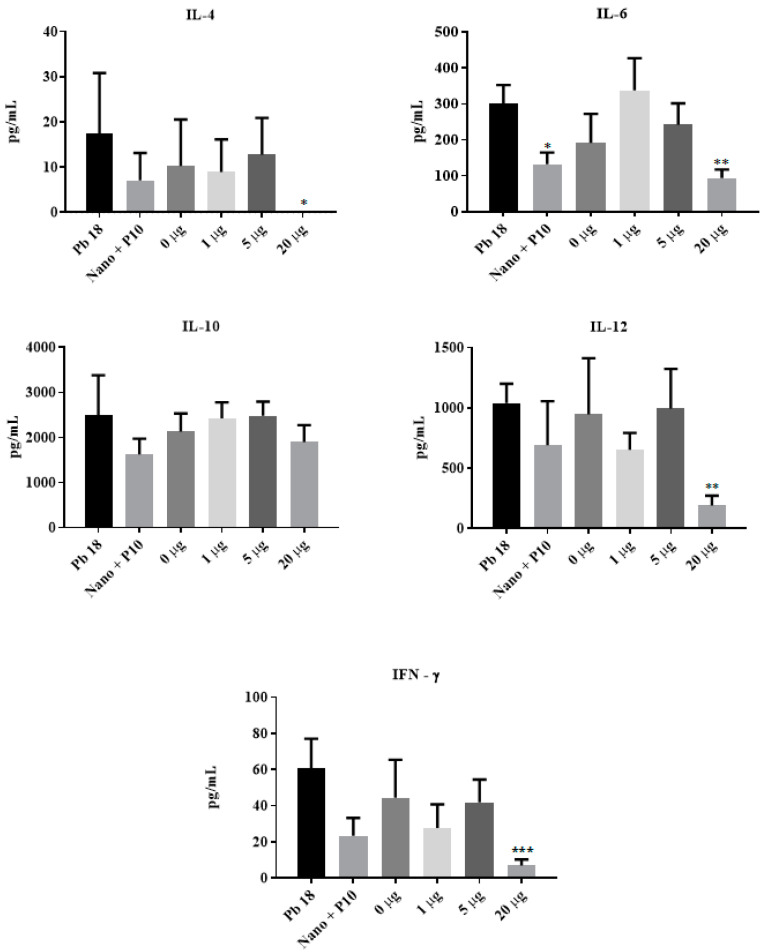
Determination of cytokine production in lung homogenat of BALB/c mice after 51 days of infection with P. brasiliensis. Mice were infected and randomly distributed in the following groups: Pb 18: non-treated; Nano + P10: co-administered vaccine (empty nanoparticles + 20 μg/10 μL free-form of P10 peptide); 0 μg: treated with empty nanoparticles; 1 μg: treated with P10-complexed nanoparticles containing 1 μg of P10/10 μL nanoparticles; 5 μg: treated with complexed nanoparticles containing 5 μg/10 μL of P10; 20 μg: treated with complexed nanoparticles containing 20 μg/10 μL of P10. The cytokines showed a tendency of mixed immune response, where the anti-inflammatory and pro-inflammatory cytokines appear to be in balance when compared to the control group (Pb 18). The group treated with nanoparticles complexed with 20 μg of P10 had a significant reduction in the cytokines IL-4, IL-6, IL-12 and IFN-γ. Data are represented as mean SEM and were analyzed by ANOVA statistical test with * *p* ≤ 0.05, ** *p* ≤ 0.01 and *** *p* ≤ 0.001 in comparison to non-treated mice (Pb18).

The cytokine profile was investigated in the mouse lung macerate by quantifying the Th1 cytokines IL-12 and IFN–γ or Th2 cytokines IL-4 and IL-6. Figure 4 shows the cytokine profile representing a diverse Th1 and Th2 immune response, which was not expected based on the type of nanoparticle used and the known immunomodulatory characteristics of the P10 peptide.

Mice that received 20 μg of P10 complexed within chitosan nanoparticles showed significant reduction in the cytokines IL-4, IL-6, IL-12, and IFN–γ. IL-6 levels were also lower in mice co-administered with empty nanoparticles and free P10. Other cytokine levels were not altered after treatment with free P10 mixed with empty chitosan nanoparticles or with lower doses of P10 encapsulated in the chitosan nanoparticles (1 and 5 µg).

### 3.5. Lung Histopathology of P10-Complexed Nanoparticles Treated Animals 

**Figure 5:**
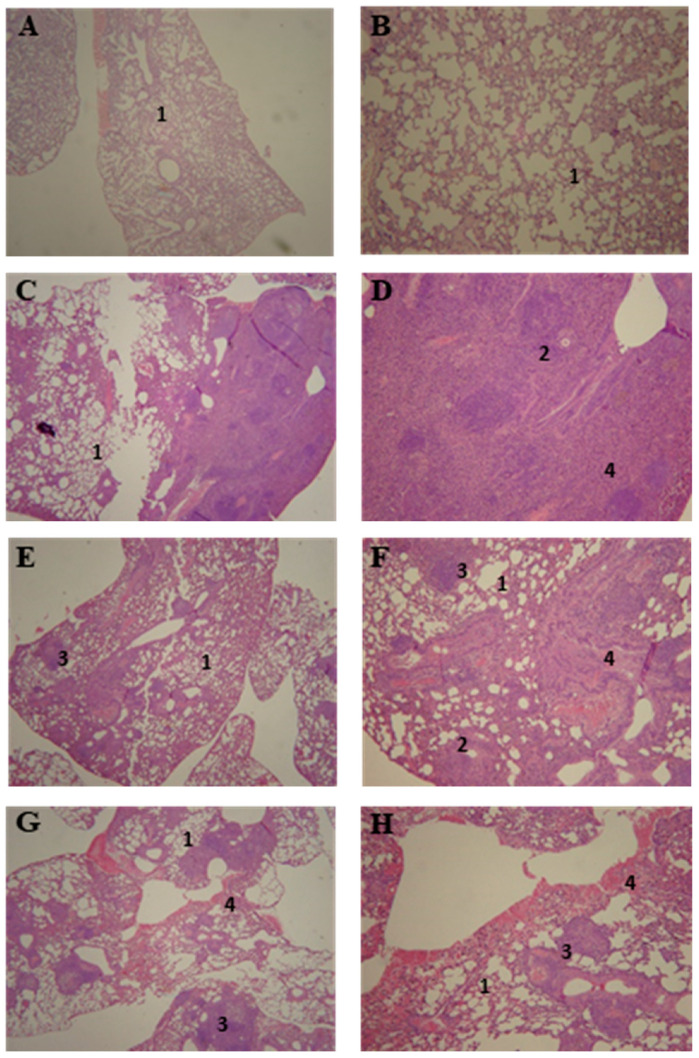

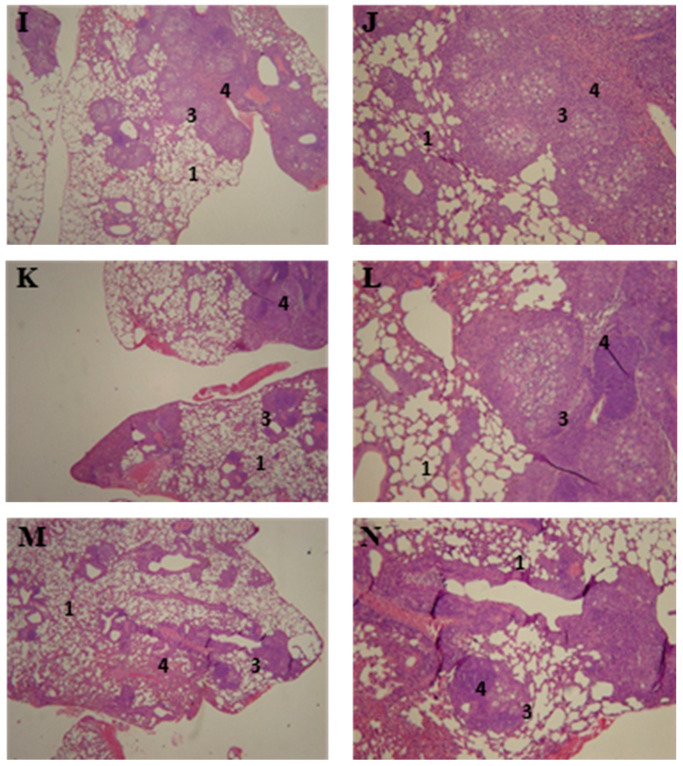
BALB/c mice lung histology (HE-stain) after 51 days of infection with *P. brasiliensis.* Findings include; (1) preserved lung tissue, (2) loose granulomas, (3) well defined granulomas and (4) presence of inflammatory infiltrates. Figures depict (**A**,**B**) non-infected mice and: infected and (**C**,**D**) non-treated, (**E**,**F**) treated with empty nanoparticles and 20 µg of free form of P10, (**G**,**H**) treated with empty nanoparticles and treated with P10-complexed nanoparticles with (**I**,**J**) 1 µg of P10, (**K**,**L**) 5 µg of P10 and (**M**,**N**) 20 µg of P10. (**A**,**C**,**E**,**G**,**I**,**K**,**M**) bear 8× magnification; (**B**,**D**,**F**,**H**,**J**,**L**,**N**) bear 20× magnification.

The lung histology of the infected and untreated mice (Figure 5C,D) showed a disorganized granuloma and inflammatory cell infiltrate. The group treated with empty nanoparticles (Figure 5G,H) presented a mixture of loose and defined granulomas. The groups treated with P10 associated with empty nanoparticles (Figure 5E,F), or P10-complexed nanoparticles at 1 µg (Figure 5I,J), 5 µg (Figure 5K,L) or 20 µg (Figure 5M,N) also presented granulomatous lesions; however, those lesions were more defined and organized. Additionally, the pulmonary structure was better preserved in these P10-treated animals when compared to the untreated group.

## 4. Discussion

The prolonged treatment time, relapses, and continuous medical follow-up that occur in PCM reveal how crucial the development of new therapeutic alternatives for the treatment of infection, in order to improve both, clinical conditions and the well-being of patients. Among these new alternatives, it is worth highlighting the use of the immunotherapy, particularly the P10 peptide, which exerts an important effect of reducing the fungal load in different forms of murine PCM [15,18].

Although the P10 peptide represents an advance in the experimental treatment of PCM, the P10 peptide itself does not have efficacy when used without an adjuvant in the animal model. While, it has strong immunomodulatory effects in cultured cells [25], improvements in this therapeutic strategy are needed. One of the approaches is to protect P10 from in vivo degradation through complexation within polymeric nanoparticles such as those made of PLGA or chitosan [18,26].

The use of nanoparticles to carry the peptide P10 was first evaluated in previous studies by our research group. P10-PLGA nanoparticles with dimercaptosuccinic acid (DMSA) were used to treat PCM in a murine model [18]. Both studies showed the capacity to reduce the fungal load and the concentration of the peptide P10 used. The advantages of our chitosan nanoparticles over PLGA nanoparticles are that it can be administered intranasally instead of intraperitoneally, and that the local delivery to the airways avoids the necessity to incorporate other molecules, such as DMSA, to induce tropism to the respiratory system.

Studies have found that using a DNA vaccine based on the heat shock protein of *Mycobacterium leprae* (HSP 65) loaded on PLGA nanoparticles or liposomes reduced the fungal load of *P. brasiliensis* [27]. In both cases, the use of PLGA nanoparticles and liposomes containing the P10 peptide or the DNA vaccine (HSP 65 of *Mycobacterium leprae*) induced an immune response with a Th1 pattern with increased production of IL-12 cytokines and or IFN-γ [18,28].

Chitosan nanoparticles have already been used as fungicides or fungistatics that cause morphological changes in cell walls and/or membranes [29,30]. Their efficacy as carriers and deliverers of molecules, such as antifungals has also been verified [31].

To the best of our knowledge, this is the first study that utilizes chitosan nanoparticles as carriers for an intranasal vaccine against fungi, although chitosan nanoparticles have already been used in intranasal vaccines against viral infections [32,33,34,35].

In this work, the P10 peptide was incorporated within chitosan nanoparticles. Physiochemical characterization indicated the size, PDI and zeta potential were suitable for in vivo administration, as suggested in other studies on the same topic [36,37]. The PDI mean value was less than 0.5, indicating a monodisperse nanoparticle size, and the positive zeta potential allows a greater interaction with the negative cell membrane [37,38,39].

In our results, the difference between the sizes of the empty nanoparticles, and the nanoparticles complexed with the P10 peptide, can be explained by the interaction of the peptide with chitosan and TPP [36]. The neutral residual charge of the P10 peptide and its hydrophobicity explains the interaction with the TPP negative charges and with the positive charge of the chitosan. The same interaction of charges also explains the reduction of the zeta potential of the complexed nanoparticles [36].

The hemolysis assays corroborate the data from literature that confirm the biocompatibility of chitosan nanoparticles with or without the P10 peptide [19,40,41]. In the cell viability assays, it was observed that after 72 h there was a cytotoxic effect caused by nanoparticles complexed with the P10. Possible explanations for cytotoxicity, include the presence of dimethyl sulfoxide, which is necessary for solubilization of P10, and the peptide’s strong immunomodulatory ability, which could lead to cell activation and death [42].

The use of the chitosan nanoparticles complexed or co-administered with the P10 peptide provided a significant reduction in the fungal load. Further, complexation in chitosan nanoparticles allowed a decrease of 4 (5 μg) to 20 (1 μg) times the usual dose of P10 peptide (20 μg) used to generate an effective immune response [18,26]. 

Complexation of nanoparticles allows a significant reduction of the P10 peptide concentration used in the vaccine, and we believe this benefit is mainly contributed by the adjuvanting effect of nanoparticles. Previous studies of our group where P10 peptide was administered with different adjuvants showed the lowest amount of P10 peptide required to elicit a productive immune response in infected mice was 20 μg [18,26].

We believe that complexed or co-administered chitosan nanoparticles act as adjuvants favoring the immune response, and reducing the needed peptide concentrations to generate this type of stimulus. [18]. No tests were carried out using empty nanoparticles with concentrations of the p10 peptide other than 20 µg/10 µL, as this result was not expected.

In this study, the results indicate that the use of chitosan nanoparticles, complexed or associated with the P10 peptide, promotes a mixed pattern of immune response with both Th1 and Th2 type cytokines. These data corroborate other studies in which authors describe that chitosan has the ability to modulate both, Th1 or Th2 and induce a mixture of Th1/Th2 cytokines [43,44,45,46]. In contrast, a previous study from our group showed modulation towards a Th1-type immune response with increased production of IL-2, IL-12 and IFN-γ and reduction of IL-4 and IL-10 [10,11,26]. The reduction of the cytokines IL-4, IL-6, IL-12 and IFN-γ during treatment with complexed nanoparticles containing 20 μg/10 μL of P10 was an unexpected result and may indicate the beginning of the process of a serological cure [3,4]. Although the timeframe chosen to assess cytokine production was based on previous experiments, 51 days post-infection and 21 days post-treatment could potentially not be the ideal time to determine modulation of cytokine production in this model. Therefore, the timepoint, use of a new kind of nanoparticle, and the route of immunization (intranasal) could account for these observations.

## 5. Conclusions

The chitosan nanoparticles presented appropriate physical and chemical characteristics for mucosal administration of P10. They allowed a 4 to 20-fold reduction in the dose of P10 peptide compared to other immunization strategies studied by our research group, and were able to promote a significant reduction in pulmonary fungal load. The treatment induced a mixed Th1 and Th2 immune response. Moreover, the histological analysis of the lungs of infected mice, treated with P10-complexed to chitosan nanoparticles, revealed more preserved structures in comparison to untreated mice.

## Figures and Tables

**Table 1:** Physio-chemical characteristics of empty NP (nanoparticles without P10) and complex NP (nanoparticles complexed with 20 μg/10 μL of P10 peptide).

Characteristics	Empty	Complexed
**PDI**	0.52 ± 0.06	0.454 ± 0.04
**Size**	338 ± 8 nm	226 ± 14 nm
**Zeta potential**	41.14 ± 1.4 mV	19.6 ± 3.5 mV
